# Hidden analyses: a review of reporting practice and recommendations for more transparent reporting of initial data analyses

**DOI:** 10.1186/s12874-020-00942-y

**Published:** 2020-03-13

**Authors:** Marianne Huebner, Werner Vach, Saskia le Cessie, Carsten Oliver Schmidt, Lara Lusa, on behalf of the Topic Group “Initial Data Analysis” of the STRATOS Initiative (STRengthening Analytical Thinking for Observational Studies, http://www.stratos-initiative.org), Dianne Cook, Marianne Huebner, Saskia le Cessie, Lara Lusa, Carsten Oliver Schmidt, Werner Vach

**Affiliations:** 1 Department of Statistics and Probability, Michigan State University, East Lansing, MI USA; 2 Institute of Medical Biometry and Epidemiology, University Medical Center, Hamburg, Germany; 3 Department of Orthopaedics and Traumatology, University Hospital Basel, Basel, Switzerland; 4 Department of Clinical Epidemiology and Department of Biomedical Data Sciences, Leiden University Medical Center, Leiden, The Netherlands; 5 Institute for Community Medicine, SHIP-KEF University Medicine of Greifswald, Greifswald, Germany; 6 Department of Mathematics, Faculty of Mathematics, Natural Sciences and Information Technology, University of Primorksa, Koper, Slovenia; 7 Institute of Biostatistics and Medical Informatics, University of Ljubljana, Ljubljana, Slovenia

**Keywords:** Initial data analysis, Reporting, Observational studies, STRATOS initiative

## Abstract

**Background:**

In the data pipeline from the data collection process to the planned statistical analyses, initial data analysis (IDA) typically takes place between the end of the data collection and do not touch the research questions. A systematic process for IDA and clear reporting of the findings would help to understand the potential shortcomings of a dataset, such as missing values, or subgroups with small sample sizes, or shortcomings in the collection process, and to evaluate the impact of these shortcomings on the research results. A clear reporting of findings is also relevant when making datasets available to other researchers. Initial data analyses can provide valuable insights into the suitability of a data set for a future research study. Our aim was to describe the practice of reporting of initial data analyses in observational studies in five highly ranked medical journals with focus on data cleaning, screening, and reporting of findings which led to a potential change in the analysis plan.

**Methods:**

This review was carried out using systematic search strategies with eligibility criteria for articles to be reviewed. A total of 25 papers about observational studies were selected from five medical journals published in 2018. Each paper was reviewed by two reviewers and IDA statements were further discussed by all authors. The consensus was reported.

**Results:**

IDA statements were reported in the methods, results, discussion, and supplement of papers. Ten out of 25 papers (40%) included a statement about data cleaning. Data screening statements were included in all articles, and 18 (72%) indicated the methods used to describe them. Item missingness was reported in 11 papers (44%), unit missingness in 15 papers (60%). Eleven papers (44%) mentioned some changes in the analysis plan. Reported changes referred to missing data treatment, unexpected values, population heterogeneity and aspects related to variable distributions or data properties.

**Conclusion:**

Reporting of initial data analyses were sparse, and statements on IDA were located throughout the research articles. There is a lack of systematic reporting of IDA. We conclude the article with recommendations on how to overcome shortcomings in the practice of IDA reporting in observational studies.

## Background

Much discussion has focused on selective reporting based on statistical significance and *p*-values in research. An overemphasis on statistical significance possibly led to spurious results in medical research [1]. However, p-values are only the “tip of the iceberg” in a long data pipeline that includes data cleaning, data screening or exploratory data analysis, before the statistical modelling takes place [2]. A typical part of this data pipeline may be referred to as Initial Data Analysis (IDA). IDA typically takes place between the end of the data collection and the start of those statistical analyses that address the research questions, although some IDA aspects may occur already during the data collection process.

A recently introduced IDA framework distinguished six IDA steps [3]. The first step is to set up the meta data, which includes all background information required to properly conduct subsequent IDA steps. In the next two steps, the data should be systematically cleaned and screened. Data cleaning aims to identify data errors and, if possible, correct them. Data screening systematically reviews and documents data properties and data quality that may affect future analysis and interpretation (step 3). Careful reporting of all relevant insights obtained from the cleaning and screening steps is needed to inform researchers who work with the data (step 4). Data properties may not conform to our subject knowledge that was used to develop the analysis plan. For example, the distribution of some variables is unexpectedly skewed, more values are missing than expected, or data errors are detected. In that case it may be necessary to refine or update the analysis plan (step 5). The final step of IDA is the reporting relevant findings of IDA in research papers to document all findings and analytic choices that impact the interpretation of results.

Wasserstein et al. [4] coined the term ATOM (**A**ccept uncertainty, be **T**houghtful, **O**pen, and **M**odest.) for good research practice. Conducting IDA can contribute to good research practice and is related to the ATOM principles. *Thoughtful* research begins with clear objectives, and these objectives are part of the meta data. Subsequent IDA steps aim to provide reliable knowledge about the data to enable responsible statistical analyses and interpretation. Reporting all relevant findings of the IDA and any update of the analysis plan which may be revealed during IDA, contributes to the necessary *open*ness in research. Furthermore, IDA may point to limitations of the data, which when reported, contribute to *accepting uncertainty*.

Completeness in reporting requires not only the description of limitations of the data, but also a description of the initial analyses performed and presenting the findings thus obtained. Yet, IDA is often “hidden” in the sense that analyses and subsequent decisions are often conducted in an unplanned and unstructured way, only partially shared among research collaborators or described in research papers. Readers may not appropriately understand the findings due to poor reporting. Failing in reporting can lead to publication bias [5] or invalid results [6].

It is reasonable to expect that not all elements of IDA will be reported in a published research article because of the large scope of IDA relative to common space restrictions. The reporting guideline for observational studies STROBE statement [7] considers some aspects of IDA reporting. This consists of the description of baseline and outcome variables or the reporting of missing values in variables and numbers of missing individuals at each stage of study. However, this may not inform the reader completely about all relevant IDA results and decisions make in the IDA steps. Our aim was to describe the practice of IDA reporting in observational studies in five highly ranked medical journals with focus on data cleaning, screening, and reporting of findings which led to updating the analysis plan. We conclude the article with recommendations on how to overcome short comings in the practice of IDA reporting in observational studies.

## Methods

This was a methodological study where the PubMed database was used to identify observational studies to review reporting practices of IDA. The review was carried out using systematic search strategies using with eligibility criteria for articles to be reviewed. Reporting adhered to the PRISMA guidelines. To aid transparency, the PubMed search strategy, data collection form, and PRISMA checklist are included in the supplement. The a priori protocol is available on the STRATOS TG3 website (https://www.stratosida.org/activities/project-systematic-review-of-ida-reporting).

### Sampling frame

Papers were selected from five medical journals *(The New England Journal of Medicine (NEJM), Lancet, Journal of Clinical Oncology (JCO), Circulation (CIRC), Journal of the American Medical Association (JAMA)*)*.* All papers published in a six-month window from January 1, 2018 to July 15, 2018 meeting the inclusion criteria were included. The primary reviewer [MH] screened the titles and abstracts against the inclusion criteria. Full reports were obtained of all articles which appeared to meet the inclusion criteria below. Each statement in a selected paper needed to be carefully evaluated regarding its relation to initial data analysis. Thus for an equal representation across journals five papers from each journal were randomly selected and reviewed by two reviewers. The sample size of 25 papers was not based on a formal sample size criterion, but was perceived as sufficient to gain general insights on IDA reporting. The random sampling protects against unforeseen selection bias. For each journal selected papers were ordered, then the order was permuted using the statistical software R, and the first 5 papers on the list were selected, to retain the equal representation across journals. If, upon examination, an article did not meet the inclusion criteria, it was replaced by the next paper on the list from the target journal.

### Inclusion criteria

- Observational study, original research articles
- Published in one of the selected journals and available between January 2018 and July 15th, 2018 (including Epub ahead of print).

### Exclusion criteria

- Clinical trials, randomized experiments, laboratory studies, genetics or genomics studies, letters, editorials, reviews, guidelines, comments
- Fewer than 50 participants
- Simulation studies, imaging studies, cost studies
- Studies published only in abstract form
- No clear research aim stated (This was necessary to separate IDA from the planned statistical analyses.)

A flow chart of study selection was created and characteristics of the included studies were summarized.

### Data extraction

Data were extracted from the selected papers using a standardized data extraction form developed for this review. An online submission form was prepared, piloted and refined prior to use by two authors LL and MH. This was based on the conceptual framework for IDA [3] which was developed for studies including a primary data collection, but major parts of the framework apply also to studies based on a secondary data analysis. The form included data on study background (author, country, sample size, data source), elements of IDA framework reported (data cleaning, screening, change in the analysis plan). Each aspect was classified by the location in the paper where the respective aspect was targeted and ranked by sufficiency of information (not mentioned, mentioned, mentioned with sufficient detail or not applicable). Text excerpts from the articles could be added in the form. Information was requested separately for the outcome variable(s) according to the main research question. Other variables were labeled as “non-outcome variables.” Information on statistical methods that were used to describe variables and their placing in the paper, was also collected. The reporting of missing values was assessed. We distinguished item missingness as data values partially missing from unit missingness, which referred to complete missingness of measurements from observational units (e.g. no observations for an individual at a certain time point).

The full articles were reviewed, and the location of IDA statements was noted as Introduction, Methods, Results, Discussion, and Supplement. If topics were mentioned in more than one section, the main selections were reported and therefore the sum of reported locations could exceed the sample size of 25 articles.

All co-authors reviewed at least five papers, with MH reviewing all papers to assure consistency in applying criteria. In this paper we report the consensus between two reviewers.

### Data analysis

Both quantitative summaries and qualitative evaluation of text excerpts were employed. Each extracted item was summarized overall and by location in the article. A summary stratified by journal was not attempted due to the small number of articles from each journal.

After the initial inspection of the extracted text excerpts it became clear that different reviewers had different interpretations of the distinction between “sufficient “and “mentioned.” It was therefore decided to collapse these terms.

The mapping of the text excerpts to one or possible multiple IDA elements was discussed in several meetings (in person or online) by all co-authors until agreement was reached.

## Results

**Fig. 1:**
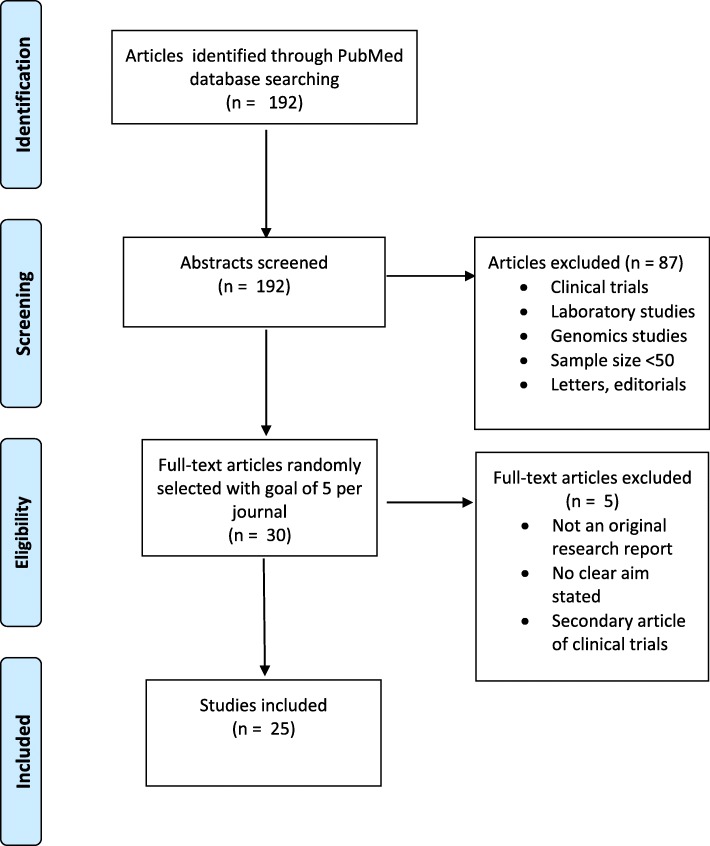
Flow Diagram for Initial Data Analysis reporting

A total of 192 candidate articles were identified in the five journals for the time period January 1 to July 15, 2018 in the five journals (Table 1). A total number of 25 articles were included in this review (Fig. 1, Table 1).

**Table 1:** Search and selection of articles

	NEJM	JCO	Lancet	JAMA	CIRC	Total
Selected papers via Pubmed search	11	63	21	29	68	192
Included according to criteria after reviewing abstract	7	22	12	19	45	105
Included according to criteria after reviewing full text article	6	21	10	19	44	100
Randomly selected for review	5	5	5	5	5	25

Data sources for these observational studies included national registries, health insurance data bases, or health records from a single or multiple hospitals or cohort studies (Table 2).

**Table 2:** Characteristics of the included studies

Study	Journal	Location	Years of participant selection^a^	Study size^a^	Data source^a^
Inohara et al. [8]	JAMA	USA	2013–2016	141,311	Stroke registry
Purnell et al. [9]	JAMA	USA	1995–2014	453,162	Transplant registry
Reges et al. [10]	JAMA	Israel	2005–2015	33,540	Multiple hospitals
Snyder et al. [11]	JAMA	USA	2006–2007	8529	Cancer registry
Yu et al. [12]	JAMA	China	2004–2008	271,217	Nationwide Biobank
Biccard et al. [13]	Lancet	25 African countries	2016	11,422	Multiple hospitals
Wood et al. [14]	Lancet	19 high income countries	1964–2010	599,912	Multiple CVD registries and a biobank
Dziadzko et al. [15]	Lancet	USA	2000–2010	1294	Single hospital and a medical registry of area residents
Zylbersztejn et al. [16]	Lancet	UK, Sweden	2003–2013	4,946,246	Hospital episode registries, birth and death registries
Gilbert et al. [17]	Lancet	UK	2013–2015	22,139	Hospital episode registry; death registry
Alexander et al. [18]	Circulation	Australia	1987–1996	80	Childhood cardio-myopathy registry
Nazerian et al. [19]	Circulation	Brazil, Germany, Italy, Switzerland	2014–2016	1850	Multiple hospitals
Pollack et al. [20]	Circulation	USA, Canada	2011–2015	2500	Resuscitation outcomes registry
Puelacher et al. [21]	Circulation	Switzerland	2014–2015	2018	Single hospital
Chao et al. [22]	Circulation	Taiwan	1996–2015	32,160	Health Insurance database
Chow et al. [23]	JCO	USA	1962–2001	13,060	Multiple hospitals
Kenzik et al. [24]	JCO	USA	2000–2011	72,408	Cancer registry and Health insurance database
Degnim et al. [25]	JCO	USA	1967–2001	669	Single hospital
Gundle et al. [26]	JCO	USA	1989–2014	2217	Single hospital
Clarke et al. [27]	JCO	USA	2003–2015	944,227	Multiple hospitals
Hoen et al. [28]	NEJM	French territories in the Americas	2016	555	ZIKV pregnancy population cohort
Amarenco et al. [29]	NEJM	Europe, Asia, Latin America	2009–2011	3356	Stroke registry
Calderon et al. [30]	NEJM	Israel	1980–2014	1,522,731	Renal registry and population cohort
Kyle et al. [31]	NEJM	USA	1960–1994	1384	Single hospital
Mead et al. [32]	NEJM	USA	2016–2017	184	ZIKV male population cohort

^a^Only the development sample size (i.e not the validation sample size) was included here or the population of main interest for the analysis (i.e. not matched populations)

Twelve of the 25 studies were based in the USA. Studies had large sample sizes (median = 11,422 participants, IQR: 1850 to 144,816). Survival endpoints (19/25) or binary outcomes (5/25) were the most common outcomes.

### Reporting of initial data analyses

#### Data cleaning

Ten out of 25 papers (40%) included a statement about data cleaning. The statements were often general as illustrated by the following examples:

- “Clinically improbable laboratory values were removed.” [10]
- “The statistical analysis was performed on the data entered, checked, if necessary corrected and validated by the centers.” [28]
- “Registrars were asked to follow-up with outside institutions in an effort to try to ensure data completeness, but actual data completeness was not measured.” [11]

No sufficient information about the nature of the problems encountered in data cleaning, or the number of records for which errors were detected and corrected was reported. Consequently, even if data cleaning was mentioned, we often know little about the process and potential impact. More details were provided, when explicitly reporting the rules for correcting data values, or reporting the range of admissible values and number of records with values outside the range in the Supplement [10]. One paper included the computer code used for data cleaning in the Supplement [20], which made the data cleaning potentially reproducible.

The information about data cleaning was reported in Methods (*n* = 5), Discussion (*n* = 3) or Supplement (*n* = 4).

#### Data screening

Data screening examines data properties that do not touch the research questions but may affect the interpretation of results from statistical models or may lead to updating the analysis plan [3]. This includes a systematic review of the distribution of variables and missing data. Understanding associations between variables can support decisions about modeling and later interpretation of the results. Statements about data screening were grouped by outcome and non-outcome variables and by location in the papers **(**Table 3**).** Methods of descriptions of such variables could include quantitative or graphical data summaries. For example,

- Variables are described by counts or averages, such as “Categorical variables are presented as number (percent); age and time from onset are presented as median and 25th through 75th interquartile range; clinical features as presented as mean ± SD.” [20]
- Description of outcome variables may refer to number of events, mean-follow-up time, or cumulative incidence functions. “Of the 61 sites, 42 had follow-up data on more than 50% of their patients at 5 years (3847 patients), who represented 80.3% of the initial cohort.” [29]

**Table 3:** Number of papers with data screening statements by location in the paper

		Location in Paper			
	Mentioned in papers, n (%)	M	R	D	S
Description of non-outcome variables	25 (100%)	5	24	0	15
Description of missing values of non-outcome variables	19 (76%)	6	12	0	6
Reporting association between non-outcome variables	14 (56%)	5	6	0	5
Description of non-outcome variables for subgroups	21 (84%)	2	19	1	11
Description of transformation of non-outcome variables	10 (40%)	4	4	0	2
Description of outcome variable(s)	25 (100%)	2	25	0	9
Information of missing values for outcome variables	12 (48%)	3	7	3	4
Description of methods for outcome variables	19 (76%)	13	4	0	1
Description of missingness of subjects	15 (60%)	1	11	2	5
Description of transformations in outcome variables	7 (28%)	1	6	0	0

*Abbreviations*: *M* Methods, *R* Results, *D* Discussion, *S* Supplement

A common aspect of data screening is the description of *non-outcome variables.* These were presented in all articles, commonly in the Results section (*n* = 24) but also in the Supplement (*n* = 15) and occasionally in Methods (*n* = 5). Most articles reported this information in tables (*n* = 21) and text (*n* = 20). Data visualizations were rarely used (*n* = 2). The statistical methods used to describe non-outcome variables were reported in 19 articles. Information about the association between non-outcome variables was included in 14 papers (56%). Information on missing values for non-outcome variables was reported in 19 papers (76%). The information appeared most often in Results (*n* = 12) but also in Methods and in the Supplement (*n* = 6 each). Ten papers provided information about distributions of non-outcome variables, which later implied a change in analysis plan. This information was provided in Results (*n* = 4), Methods (*n* = 4) and in the Supplement (*n* = 2). This referred mainly to categorizing non-outcome numerical variables. Some studies reported categories with small frequencies, which led to a sparser grouping than originally intended [27, 29]. In one study [8], the adequateness of a non-outcome variable was checked in the IDA. “Comparison of the multilevel model to a non-multilevel model (likelihood-ratio test) indicated a significant clustering effect of testing intensity by facility (*P*< .001). […] Therefore, the [observed/expected] ratio for each facility was calculated based on the sum of the individuals from that facility. The facility was categorized into high intensity or low-intensity categories for comparison.” [11]. However, it remained unclear to which degree the variable definition was preplanned and what the action would have been, if the likelihood ratio test had not been significant.

Data screening statements for *outcome variables* were included in all articles, and 72% (*n* = 18) indicated the methods used to describe them. Item missingness was reported in 11 papers (44%), unit missingness in 15 papers (60%).

#### Changes in the analysis plan

Eleven papers (44%) mentioned some changes in the analysis plan. Reported changes referred to missing data treatment, unexpected values, population heterogeneity and aspects related to variable distributions or data properties (Table 4). The reporting of such changes could be found in all sections of the paper except in the Introduction.

**Table 4:** Number of papers with changes of the analysis plan statements by location in the paper

Reasons for change	Number of papers, n (%)	Location in Paper			
		M	R	D	S
Unexpected Values	2 (8%)	2	0	1	0
Heterogeneity	1 (4%)	0	1	0	0
Unexpected confounding	2 (8%)	1	1	2	0
Variable Distribution	4 (16%)	3	1	1	0
Other Data Properties	2 (8%)	2	0	0	0
Missing Data	5 (20%)	4	1	1	0

*Abbreviations*: *M* Methods, *R* Results, *D* Discussion, *S* Supplement

Changes were described as follows:

1. Due to variable distributions categories of the variables were grouped, or numerical variables were categorized based on findings from IDA.
  “Because few women were underweight (1.2%), we combined underweight with normal BMI (normal/underweight) and performed a sensitivity analysis excluding the underweight group.” [27]
  Chow et al. resolved classification problems of patients by using the category with lower value. “If insufficient information was available to distinguish between grades, the lower grade was applied.” [23]
  Gilbert et al. observed that “patients had Hospital Frailty Risk Scores ranging from 0 to 99, but this was heavily skewed to the right” and categorised it using three risk levels [17].
2. Revising the planned statistical model and including additional variables due to unexpected confounding was the result of IDA in some papers.
  In the discussion, Reges et al. acknowledged that “There was a higher proportion of low SES among nonsurgical patients after matching. Given the higher mortality among low SES patients in general, SES could have been a confounder. This and other potential confounding characteristics were adjusted for in the models.” [10]
  Pollack et al. adjusted their analysis for potential confounders. “For example, bystander AED shock was more likely to receive bystander CPR, so we adjusted for this covariate in the analysis,” acknowledging that obseved differences in survival could not be attributed solely to the type of help recieved by patients [20].
3. Inclusion and exclusion criteria were modified thus leading to a change in the study population due to unexpected values or population heterogeneity.
  Biccard et al. substantially relaxed the inclusion criteria as “more than half the countries in our study could not fulfill the protocol requirements for an included sample, and in hindsight these rules were inappropriately strict despite formal acceptance by the national leaders of these requirements before the study began.” [13].
  Yu et al. exluded from the analyses the “participants from Zhejiang (*n*=56,813) where heating was rarely reported (0.6%).” [12]
4. Methods to handle missing data in the analysis or inclusion/exclusion criteria were updated.
  Snyder et al. used multiple imputation for two non-outcome variables for which they had observed more than 5% missing values. “Two variables, perineural invasion and lymphovascular invasion, had more than 5% missing values. Multiple imputation by chained equations was used to substitute predicted values for missing values with 20 imputed values.” [11]
  Amarenco et al. excluded data from some study sites, and performed subgroup analyses, some of which were not prespecified. “Sites with follow-up data on more than 50% of their enrolled patients at 5 years were selected for the analysis in this report, and all reported results pertain to this selected cohort.” [29]
  Zylbersztejn et al. used data screening to exclude hospitals with low quality data: “We excluded hospitals with high proportions of missing data or evidence of linkage error to address incomplete recording of risk factors at birth. We included hospitals with more than 500 births a year, with high completeness of recorded birthweight and gestational age, and hospitals where at least half of all deaths were linked to a death certificate”, and “We developed criteria for identifying hospitals with high completeness of gestational age and birth weight, and high quality of linkage with ONS mortality data in an iterative process.” [16]
5. Other data properties may influence statistical models.
  Wood et al. exluded from combined analyses of several data sources “studies with fewer than five incident cases of a particular outcome” to avoid model overfitting [14].

#### Sensitivity analyses

Sensitivity analyses are commonly used when checking on robustness of models and conclusions. These are often pre-planned in the study design phase, but could be a consequence of IDA and planned before the main analyses instead of having to rely on post hoc analyses. For example,

- “Because few women were underweight (1.2%), we combined underweight with normal BMI (normal/underweight) and performed a sensitivity analysis excluding the underweight group.” [27]
- Inclusion criteria were relaxed during the data collection process and it was noted that “Before analysis we therefore decided to present the data describing the full cohort, and include a per-protocol analysis of the predefined representative sample for comparison.” [13]
- “Event rates were estimated among the overall study sample (main analysis), among patients evaluated by a stroke specialist within 24 hours after symptom onset (prespecified sensitivity analysis), and among patients from the 33 sites with follow-up data on more than 80% of their patients at 5 years (post hoc sensitivity analysis)” [29]

We point out that it was sometimes difficult to decide whether an information about a certain action reflected a consequence of IDA or had been preplanned. For example, the statement “If insufficient information was available to distinguish between grades, the lower grade was applied.” [23] may reflect a rule developed during IDA, but it may also reflect a rule already decided on in the study protocol.

## Discussion

Our aim was to describe the practice of reporting in observational studies in highly ranked medical journals. A total of 25 papers about observational studies from five journals (Circulation, JAMA, JCO, Lancet, NEJM) were reviewed. The selected papers included data from disease registries, health insurance data bases, or electronic health records from single or multiple hospitals and cohort studies. To separate IDA from the planned statistical analyses, the research aim for each article was identified as the first step in the review.

This literature review shows that there is only a fragmented reporting of IDA. Only 40% of the articles included a statement on data cleaning. Such statements could be found in the methods or results section, or in the supplement. Only one paper made the data cleaning process reproducible by providing computer code. In contrast, in genomic studies, reporting of data cleaning is standard practice, e.g. call rate, criteria for linkage disequilibrium, sample quality, and how many samples or variables are excluded during this process [33]. An inspection of the data sources of the studies revealed that many studies did not perform a primary data collection but were based on analyzing existing data. This may limit the need to conduct IDA as part of the current study as parts of the IDA may have been completed prior to the study and may hence decrease the likelihood of reporting on IDA in the paper. However, when no information about data cleaning is given, the reader is unsure whether the authors have assured themselves of all relevant data properties. Ideally, authors should report, what percentage of data needed corrections or a confirmative that no major data cleaning was needed.

Some of the recommendations in the STROBE statement, related to data screening, were included in the articles, such as a description of the characteristics of study participants and summarizing outcome events or follow-up times. While all articles included a table or a description of participant characteristics, sometimes with additional information in the supplement, there were few comments on whether these findings conformed to expectations about the population. Only 76% of the papers reported item missingness. Some variables of interest were described for subgroups defined by another variable (this was labeled “association between non-outcome variables” in Table 3). We observed that, other than descriptions of subgroups, there were almost no studies who reported on associations between two covariates in a regression model. Quantifying the strength of associations could be relevant, for example, to support the interpretation of results from these models, or may assist in finding redundancies.

Data description by visualization was uncommon. Numerical variables were often categorized, and sometimes sparse categories were grouped, but it was difficult to infer whether these categorizations were preplanned or a consequence of IDA.

There can be insights from IDA that can lead to changing or appending the analysis plan with additional, planned analyses rather than identifying problems later during the statistical modeling process. For example, IDA may lead to additional sensitivity analyses. This shows how useful IDA can be since such analyses can then be planned before the start of the formal intended statistical analyses. Otherwise they would appear as post hoc analyses performed after seeing the results of the main intended analyses, which would diminish their value.

The placement of IDA statements varied over different sections in the articles. In our review data cleaning, data screening, and updating the analysis plan were found in all sections of the articles except the Introduction. The Discussion typically included a paragraph on limitations where some statements could be interpreted as conclusions of data screening.

A systematic process for IDA and its reporting is lacking [3]. This is a review of papers from highly ranked medical journals with reporting check lists and a rigorous statistical review process. It is possible that in lower tier journals the IDA reporting is different and may depend on whether a study protocol is required that includes a careful analysis plan.

Such a process and clear reporting of the findings would help to understand the potential shortcomings of a dataset, such as missing values, or subgroups with small sample sizes, or shortcomings in the collection process, and to evaluate the impact of these shortcomings on the research results. IDA allows the researcher and domain expert to become more familiar with the data, and can inform, for example, about data quality issues ideally already during the data collection process. A clear reporting of findings is also relevant when making datasets available to other researchers. Initial data analyses can provide valuable insights into the suitability of a data set for a future research study [34, 35].

### Limitations

There are limitations to this study. First, this review was limited to 25 papers in medical journals. However, the aim was to get a general impression of IDA reporting with examples across five medical journals and a discussion on how reporting might be improved. We did not find differences in reporting between the journals. Second, IDA in studies based on disease registries, large electronic health record data bases, or population cohorts may have been performed prior to the study leading to less IDA reporting. Third, it was difficult to determine whether analyses were preplanned or were part of IDA. To alleviate this problem there were two reviewers for each article, and one person reviewed all articles to make sure criteria were consistently applied.

## Conclusions

Reporting of initial data analyses in research publications is sparse, and statements on IDA are located throughout the research articles, illustrating the lack of any systematic reporting of IDA. Recommendations to improve the poor practice can be made, but a full consensus of what should be expected of IDA reporting needs to be developed. Challenges exist for multi-purpose studies, combining different data sources, or reusing existing data [3].

We present some thoughts towards how IDA reporting could be improved in Table 5.

**Table 5:** Recommendations for reporting practice for initial data analyses

	Current reporting practice	Recommendations for improved reporting practice
1	Information on IDA is sparse and may suffer from selective reporting	Full reporting of relevant results as supplementary material and reporting of all results with impact on analysis/interpretation in the paper
2	Information on IDA can be found in all sections of a paper.	• IDA methodology to be described in Methods; • IDA results to be described in Methods or Results; • Impact of IDA on interpretation to be described in discussion.
3	Distinction between pre-planned decisions and IDA-driven decisions are unclear.	Pre-planned decisions should be reported in Methods; IDA driven alterations of the analysis plan should be reported with motivation in Methods.
4	Characteristics of participants are listed without comments.	Participants’ characteristics should be checked for consistency with expectations and for potential impact on analysis and interpretation. At a minimum a statement should be included to confirm no violated expectations.
5	Reporting on missingness is incomplete.	Full reporting of missingness, e.g. a flow chart describes unit missingness and a table for item missingness of variables
6	Associations among variables are not reported.	Associations not involving the research question but with potential impact on interpretation of results should be reported

Following these recommendations would be an important step towards a more transparent and systematic reporting of analyses which are so often hidden.

## Supplementary information

**Additional file 1.** Data collection form.

**Additional file 2.** PubMed search terms.

**Additional file 3.** PRISMA check list.

## Data Availability

We provided as supplementary information the data collection form (Additional file 1), PubMed search terms (Additional file 2), and PRISMA checklist (Additional file 3).

## Abbreviations

IDA: Initial Data Analysis
PRISMA: Preferred Reporting Items for Systematic Reviews and Meta-Analyses
STRATOS: Strengthening Analytical Thinking for Observational Studies
STROBE: Strengthening the Reporting of Observational Studies in Epidemiology
