# Supplementary material for: Hidden analyses: a review of reporting practice and recommendations for more transparent reporting of initial data analyses
Source: BMC Med Res Methodol. 2020 Mar 13;20:61. doi: 10.1186/s12874-020-00942-y (PMC7071755; doi:10.1186/s12874-020-00942-y)
Supplement: Supplementary file 1 — Additional file 1. Data collection form. [file 12874_2020_942_MOESM1_ESM.docx]

**Data Collection Form: Initial Data Analysis Literature Review**

| **Study details** |  | | |
| --- | --- | --- | --- |
| Reference |  | Journal |  |
| Was a statistician a co-author? | Yes  No |  |  |
| Years of patient selection |  | Countries |  |
| Number of study centers |  | Sample size |  |
| Data source | *e.g. single hospital, registry* | | |

| **Aims of the Study** | *Statement of aim* |
| --- | --- |
| Model(s) for the primary aim | GLM  Survival  Other |
| Main statistic of interest | OR  HR  Other |

| **Data Screening** | | |
| --- | --- | --- |
| **Item** | **Answer*** | **Text excerpts** (Examples) |
| Was a statement about data cleaning included? | yes-no | *“Data quality checks were performed.”*  *“Variables with more than 10% missing data were …”* |
| Was a patient flow diagram (STROBE) included? Or was patient inclusion/exclusion adequately described in the text? | 0-1-2  Location |  |
| **Non-outcome variables** |  |  |
| Was there a description of methods? | 0-1-2  Location | *“Continuous variables were summarized with means and standard deviations.”* |
| Was a description presented in the text? | 0-1-2  Location | e.g. data summary of a few selected variables |
| Was a description presented in a table? | 0-1-2  Location | Table of patient characteristics with missing data for variable |
| Was a description presented in a figure? | 0-1-2  Location | e.g. histogram, bar charts, or boxplots |
| Did this include item missingness? | 0-1-2  Location | n (%) for each variable, reasons for missingness |
| Description of non-outcome variables for subgroups? | 0-1-2  Location |  |
| Were associations between non-outcome variables included in the text? | 0-1-2  Location | e.g. correlations |
| Were associations between non-outcome variables included in a table | 0-1-2  Location | e.g. table of characteristics stratified by groups |
| Were associations between non-outcome variables included in a figure | 0-1-2  Location | e.g. scatter plot |
| Were there transformations for non-outcome variables? (not pre-planned) | 0-1-2  Location | e.g. categorization, log-transform |
| **Outcome variables** |  |  |
| Was there a description of methods? | 0-1-2  Location | *“Cumulative incidences were calculated …”* |
| Was a description presented in the text? | 0-1-2  Location | e.g. Mean follow-up, number of events |
| Was a description presented in a table? | 0-1-2  Location | e.g. mean (standard deviation) for continuous outcomes |
| Was a description presented in a figure? | 0-1-2  Location |  |
| Did this include item missingness? | 0-1-2  Location | n (%) for each variable, reasons for missingness |
| Were there transformations for outcome variables? (not pre-planned) | 0-1-2  Location | e.g. categorization, log-transform |
| Was unit missingness reported? | 0-1-2  Location |  |
| **Repeated Measures** |  |  |
| For repeatedly measured variables is the frequency of missingness described? | 0-1-2-NA  Location | e.g. proportion of missingness over time |
| Are data properties described for cluster variables? | 0-1-2-NA  Location | e.g. centers, years, instruments |
| **0=not mentioned, 1=mentioned, 2=sufficient detail, NA=not applicable.*  *Location: introduction, methods, results, discussion, supplement* | | |
|  | | |
| **Refining or updating the analysis plan** | | |
| **Item** | **Answer*** | **Text excerpts** (Examples) |
| Was there a change in analysis plan noted in the paper? | 0-1-2-NA  Location |  |
| Due to unexpected values | 0-1-2-NA  Location | e.g. exclusion of variables or of subjects |
| Due to distribution of a variable | 0-1-2-NA  Location | e.g. needing transformation |
| Due to data properties | 0-1-2-NA  Location | e.g do not fulfill requirements of model |
| Due to missing data | 0-1-2-NA  Location |  |
| Due to unexpected population heterogeneity | 0-1-2-NA  Location | e.g. leading to subgroups, stratification |
| *0=cannot be determined, 1=yes, but impact not stated, 2=sufficient detail, NA=not applicable*  *Location: Introduction, methods, results, discussion, supplement* | | |
