# Supplementary material for: Hidden analyses: a review of reporting practice and recommendations for more transparent reporting of initial data analyses
Source: BMC Med Res Methodol. 2020 Mar 13;20:61. doi: 10.1186/s12874-020-00942-y (PMC7071755; doi:10.1186/s12874-020-00942-y)
Supplement: Supplementary file 2 — Additional file 2. PubMed search terms. [file 12874_2020_942_MOESM2_ESM.docx]

**PubMed Search (July 2018)**

((((Cohort Studies[Mesh]) OR (prospectiv*[tiab] OR retrospectiv*[tiab] OR cohort*[tiab] OR "observation study"[tiab] OR observational[tiab] OR longitudinal*[tiab] OR followup[tiab] OR "follow-up"[tiab] OR followups[tiab] OR "follow-ups"[tiab] OR "followed up"[tiab])) NOT ((Case-Control Studies[Mesh:noexp]) OR (case control*[tiab] NOT medline[sb]) OR (Meta-Analysis[pt]) OR ((meta-analy*[ti] OR metaanaly*[ti]) NOT medline[sb]) OR (Review[pt]) OR (review[ti] NOT medline[sb]) OR (Randomized Controlled Trial[pt]) OR (Random Allocation[Mesh] OR Randomized Controlled Trials as Topic[Mesh]) OR (((randomized[tiab] OR randomised[tiab]) NOT (non randomized[tiab] OR non randomised[tiab])) OR randomly[tiab] OR random allocation[tiab] OR randomization[tiab] OR randomisation[tiab] OR phase[tiab]) OR (letter[pt] OR comment[pt] OR editorial[pt]) OR ((letter[ti] OR comment[ti] OR editorial[ti]) NOT medline[sb]))) AND (2018[dp])) AND (JOURNAL)

In above search terms, replace (JOURNAL) by one of the following:

("The New England journal of medicine"[Journal])

("Journal of clinical oncology : official journal of the American Society of Clinical Oncology"[Journal])

("Lancet (London, England)"[Journal])

("JAMA"[Journal])

("Circulation"[Journal])
